# Posterior Tibial Artery Blood Flow Velocity Is Increased in Patients with Plantar Heel Pain

**DOI:** 10.3390/jcm13113153

**Published:** 2024-05-28

**Authors:** Fumiya Kaneko, Sho Katayama, Shintarou Kudo

**Affiliations:** 1 Inclusive Medical Science Research Institute, 1-26-16 Nankokita, Suminoe Ward, Osaka 559-8611, Japan; kaneko-fumiya@ar-ex.jp; 2 Department of Rehabilitation, Meidaimae Orthopedic Clinic, 1-38-25, Matsubara Setagaya Ward, Tokyo 156-0043, Japan; katayama-syo@ar-ex.jp; 3 AR-Ex Medical Research Center, 4-13-1, Todoroki Setagaya, Tokyo 158-0082, Japan

**Keywords:** blood flow velocity, heel, pain, tendinopathy, ultrasonography

## Abstract

**Background/Objectives**: This study aimed to investigate the relationship between posterior tibial artery blood flow velocity and plantar heel pain (PHP). **Methods**: The PHP group comprised patients diagnosed with plantar fasciitis with plantar heel pain during gait, and the control group comprised healthy participants without plantar heel pain. The peak systolic velocity of the posterior tibial artery was measured using ultrasonography; it was measured three times on each side, and the mean value was calculated. Receiver operating characteristic curve analysis was performed to calculate the peak systolic velocity cutoff value for plantar heel pain. **Results**: 23 patients (age 58.0 ± 16.5 years; 13 males and 10 females) and 23 healthy participants (age 51.3 ± 17.3 years; 10 males and 13 females) formed the PHP and control groups, respectively. Peak systolic velocity on the affected side was significantly greater in the PHP group (44.1 ± 13.1 cm/s) than in the control group (32.7 ± 5.9 cm/s). No significant difference was observed between the left and right sides in the PHP (7.1 ± 9.8 cm/s) and control (3.7 ± 3.0 cm/s) groups. A cutoff value of 38.2 cm/s was observed on the affected side. **Conclusions**: We quantified the increase in posterior tibial artery blood flow velocity in patients with plantar heel pain. Peak systolic velocity measurements can aid in quantitatively evaluating these patients. This study was registered as a clinical trial (UMIN000046875) on 1 October 2021.

## 1. Introduction

Plantar heel pain (PHP) is a common chronic foot disease reported to be intractable, with symptoms lasting 725 days, a 10-year persistence rate of approximately 45.6%, and a relapse rate of approximately 52.9% [1]. Furthermore, PHP is one of the most common injuries in runners [2]. The symptoms of plantar fasciitis, including PHP, are reportedly worse in the morning and after long periods of rest [3]. It has become increasingly apparent that the pathological basis of tendinopathy pain is associated with elevated intratendinous resting pressure and heightened intratendinous dynamic pressure [4]. However, the precise cause of tendon pathology remains unclear. Therefore, investigating the cause of the PHP crisis is critical and will provide useful information for evaluation and treatment.

Image evaluation is essential for understanding the disease state of PHP. In a review of imaging findings [5], thickening of the plantar aponeurosis, hypoechoic and fluid accumulation in the plantar aponeurosis, thickening of the subcalcaneal fat pad, and presence of calcaneal spurs were associated with PHP. However, a thinning of the plantar aponeurosis does not significantly change the pain score [6]. Therefore, quantitative assessments that reflect the degree of pain and symptoms require further investigation.

Recently, blood flow velocity was related to pain in the lower back [7], as well as shoulder [8] and knee joints [9], discovered through ultrasound diagnostic imaging, which is a non-invasive method of examining the body internally. When tissue damage occurs, the body increases its blood flow rate, increasing blood flow to the injured area and promoting tissue healing. Thus, by assessing the blood flow velocity, the healing response at the site of pain can be confirmed.

Ultrasonography evaluation in PHP research is being increasingly used [10]. Patients with PHP develop hypoechoic areas or collections of fluid around the plantar aponeurosis [11] and have abnormal blood flow [12]. Thus, when PHP is generated, the blood flow to the sole of the foot increases, and fluid component storage may occur. The posterior tibial artery (PTA) is the most common vessel that supplies blood to the plantar area. However, the relationship between PHP and the PTA blood flow velocity is not yet understood. This study aimed to determine the blood flow velocity of the PTA in patients with plantar tendinitis and PHP and to investigate the relationship between them. The hypothesis of this study was that the PTA blood flow velocity in the PHP was significantly higher than that in the healthy foot.

## 2. Materials and Methods

### 2.1. Participants

Patients with PHP during gait and who were diagnosed with plantar fasciitis between September 2022 and June 2023 were recruited to participate and included in the PHP group. The inclusion and exclusion criteria were as follows:

Inclusion criteria

1. Having PHP when walking;

2. Pain on the first step in the morning;

3. Patients must have subjective symptoms for at least 3 months;

4. Positive heel tenderness, windlass test, dorsiflexion/abduction test, or Tinel signs.

Exclusion criteria

1. PHP less than 3 months;

2. Diabetes mellitus;

3. Gout;

4. Rheumatoid arthritis;

5. History of foot fracture or surgery.

Participants without PHP were included in the control group. The control group was recruited by age, matching those who came to the orthopedics clinic with upper limb diseases. Data on age, height, weight, body mass index, brachial systolic blood pressure, and duration of illness were obtained from their medical records. Patient-based outcome assessments included the numeric rating scale (NRS) during walking, the Foot and Ankle Ability Measure (FAAM) questionnaire—where the FAAM assessed the ADL and the Sports subscales—and PTA flow velocity. These evaluations were conducted to compare the PHP group and control group and to validate the effects of pre- and post-treatment. In the PHP group, these assessments were performed at the start of treatment and 1 month later. The institutional review board of the Morinomiya University of Health Sciences Ethics Review Committee approved this study (UMIN000046875, 2021-095) on 10 February 2022. Written informed consent was obtained from all participants.

### 2.2. Settings of the Measuring Equipment

The Noblus ultrasound system (Hitachi Medical Corporation, Tokyo, Japan) was used to obtain measurements according to a previously published method [9]. Measurements were obtained using the brightness mode (B-mode), color Doppler, and pulse Doppler methods and a 4–8-MHz linear probe. One examiner, a physical therapist with more than 2 years of experience in echography, performed the measurements.

### 2.3. Measuring PTA Blood Flow Velocity

**Figure 1:**
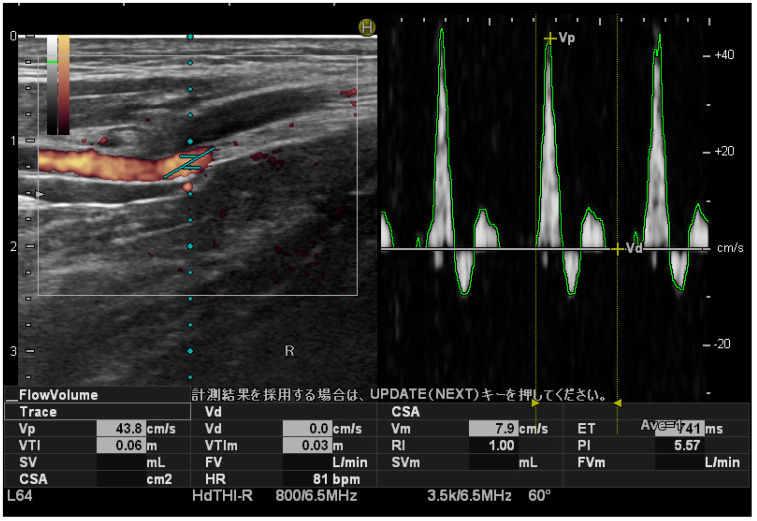
Peak systolic velocity measurement of the posterior tibial artery. Ultrasound images. Pulse Doppler sonograms of the posterior tibial artery confirmed that the blood flow had a three-phase waveform. The slant angle is adjusted manually.

The PTA blood flow velocity was measured with the participant in a supine position on the bed, and the inside and outside malleolus of the foot juxtaposed approximately 5 cm from the edge of the bed. During imaging, the PTA was identified using the power Doppler method in the short-axis image at a position where the medial malleolus was visible in the B-mode. The peak systolic velocity (PSV) of the PTA was measured using the pulse Doppler method at a slant angle of 20° and an incidence angle of 60° in the blood flow direction relative to the direction of the ultrasound beam (Figure 1). The PSV was measured three times, and the average value was calculated. Measurements were performed bilaterally.

To determine intra-rater reliability, one examiner (with over 2 years of echography test experience) performed the measurements on five healthy adults. The examiner performed the measurements with sufficient practice. The intraclass correlation coefficient (1.3) was 0.91 (minimum detectable difference with 95% confidence, 2.23). In this study, according to the classification of Landis and Koch [13], the intra-rater reliability of the measurement of PSV was good.

### 2.4. Interventions

After being diagnosed with PHP, patients in the PHP group received various treatment regimens once a week for 40 min. The interventions for the PHP group included selected conservative therapies that have been shown to be effective for PHP in previous reports. Specifically, physiotherapy consisted of stretching the plantar fascia [14], training the plantar intrinsic muscles [15,16], strength training the plantar flexor ankle musculature [17,18], taping [19,20], and ultrasound therapy [21]. Extracorporeal shock wave therapy was also administered to patients who desired it. No oral medication or steroid injections were administered. Extracorporeal shock wave therapy has been shown to be highly effective [22], so the therapists encouraged patients to receive it. However, upon request, the patient was allowed to decide whether to undergo extracorporeal shock wave therapy. Although PRP therapy has been shown to be effective for the treatment of PHP [23], it was not used in this study. The control group did not receive ankle joint intervention, nor was there a one-month follow-up.

### 2.5. Follow-Up Investigations

Patients in the PHP group were followed up for 1 month after diagnosis; the PSV, FAAM ADL, FAAM Sports, and NRS scores were the final assessments.

### 2.6. Statistical Analysis

A sample size test using G*Power (version 3.1.9.7; Heinrich-Heine-Universität Düsseldorf, Düsseldorf, Germany; http://www.gpower.hhu.de/ (accessed on 27 May 2024)) was conducted before the study. The sample size required to apply the Student’s *t*-test, which was calculated to be at least 21 patients in each group when α = 0.05, power = 0.8, and the effect size = 0.8. The figures for each factor regarding this pre-test are shown to be valid [24]. The Shapiro–Wilk test was performed to determine the normality of the results for patient information (including age, height, weight, and body mass index), as well as the PSV and scores for the NRS during walking and FAAM. The Student’s *t*-test was performed to identify the group differences in patient information (including age, height, weight, and body mass index) and the difference between the right and left sides of the PSV; the chi-square test for independence was performed for sex. Analysis of variance with the Games–Howell post hoc test was used to compare the PSV between the PHP and control groups and the differences in PSVs within the same group. When the PSV was compared between the PHP and control groups, the PSV of the right foot in the control group was used as a representative value. Correlations between the PSV and NRS score, FAAM score, and blood pressure were examined using Pearson’s correlation coefficient. Receiver operating characteristic curve analysis was performed to calculate the PSV cutoff value for PHP. The significance level was set at 5%. All statistical analyses were conducted using R4.0.2 (CRAN, freeware).

## 3. Results

### 3.1. Participants’ Information

Between September 2022 and June 2023, 23 patients were diagnosed with plantar fasciitis with PHP during gait and recruited to our study for the PHP group. Twenty-three healthy people were recruited for the control group. Table 1 shows the physical characteristics of the PHP and control groups. The mean duration of disease in the PHP group was 27.1 ± 31.4 weeks.

### 3.2. Comparison of PSV between Groups and Differences between the Left and Right Sides

**Figure 2:**
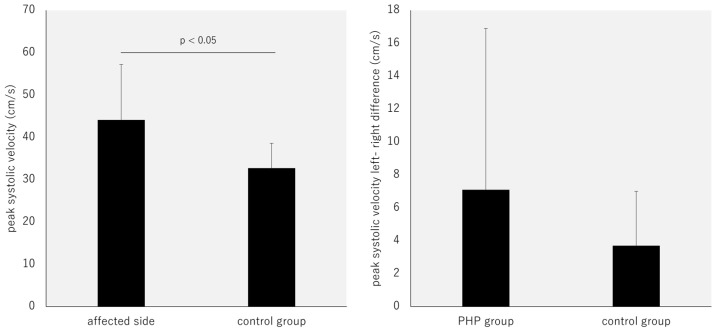
Comparison of PSV between groups and differences between the left and right sides.

The affected-side PSV in the PHP group was significantly higher than that in the control group (affected side: 44.1 ± 13.1 cm/s; control group: 32.7 ± 5.9 cm/s; *p* < 0.05, effect size: 0.50). No significant difference was observed between the two groups regarding the left–right difference (PHP group: 7.1 ± 9.8 cm/s; control group: 3.7 ± 3.3 cm/s; *p* = 0.12, effect size: 0.29) (Figure 2).

### 3.3. Correlation between PSV and Other Factors

Table 2 shows the correlations between the PSV and each factor in PHP. In PHP, no significant correlations between the PSV and NRS, FAAM ADL, FAAM Sports scores, or systolic blood pressure were observed.

Table 3 shows the numerical values and amount of change in scores before and after the 1-month follow-up. The PHP group’s improvements after one month of treatment are summarized for the PSV, NRS Score, FAAM ADL score, and FAAM Sports score. The values and modifications in each index before and after the intervention are detailed. Table 4 shows the correlation between the change in the PSV and the change in each factor after 1 month of treatment in the PHP group. The correlation between the change in PSV and the change in NRS score, FAAM ADL score, and FAAM Sports score is depicted in this table. A total of 18 individuals were successfully tracked in the 1-month follow-up survey. Five patients withdrew and were not available for follow-up. A significant moderate positive correlation was observed between the change in PSV and the change in the NRS score.

### 3.4. Cutoff Value of the PSV

The unilateral cutoff value was 38.2 cm/s (area under the receiver operating characteristic curve = 0.74, sensitivity = 65.2%, and specificity = 82.6%).

## 4. Discussion

The results of the current study showed that the PSV of the affected side of patients with PHP was significantly higher than that of the control group. After 1 month of treatment, a significant moderate positive correlation was observed between the changes in PSV and the NRS score. In contrast, no association was found between the PSV and FAAM ADL or FAAM Sports scores. The cutoff value for the PSV on the affected side was determined to be 38.2 cm/s.

According to the image evaluations of plantar fasciitis in patients with PHP, the plantar fascia thickens to ≥4 mm [25,26], the low echoic region or storage of the fluid component occurs around the plantar fasciitis [26], and abnormal blood flow occurs [12,27]. Abnormal angiogenesis occurs at the site of injury in chronic musculoskeletal diseases [28,29], and the blood flow velocity in the vessels in the area increases [8,9]. To the best of our knowledge, no research has evaluated the blood flow velocity of the PTA. Hence, the increase in blood flow velocity of the PTA in patients with PHP was quantitatively shown for the first time in this study.

The mechanism of increased blood flow velocity in plantar fasciitis and other tendon disorders is as follows. Tendon disorders, including plantar fasciitis, are commonly triggered by compressive overload [4]. This stress occurs at the tendon attachments and is exacerbated when tendons are subjected to tensile loading under compressive loading stress owing to bony prominences [30]. In addition, compressive loading stress causes the accumulation of glycosaminoglycans at the origin of the plantar fascia [31], which leads to tendon swelling and increased internal pressure [4]. At the origin of the plantar fasciitis in PHP, there is an increase in glycosaminoglycans, which promotes a neovascular response [31]. The vasculature of the lesioned tendon cells is composed of vessels with narrow inner diameters [32] and is in a constant state of hypoxia [33]. In addition, ultrasound imaging findings show increased blood flow in the microvasculature of the plantar tendon membrane in PHP [34]. Thus, neovascularization is produced in injured or diseased tendon tissue but is hypoxic. It is possible that blood flow velocity is increased to promote tissue healing in injured or diseased tendon tissue areas.

In this study, there was no correlation between the PSV and NRS or FAAM subscale scores at baseline (Table 2), and the degree of change in PSV was moderately positively correlated with the NRS score after one month (Table 4). The baseline scores showed no association with pain, but there was a correlation between the amount of change in pain and the amount of change in PSV with the intervention. This suggests that PSV may reflect the status of tissue healing in the PHP. As mentioned previously, blood flow velocity increases to contribute to tendon tissue healing [34]; therefore, it is likely that blood flow velocity would be reduced if pain was reduced. The PSV in the PHP can be used to infer the healing state of the tendon by examining the changes over time.

The correlation between the amount of change in PSV and the amount of change in the NRS at 1 month and the lack of correlation between the amount of change in PSV and the amount of change in the FAAM subscale has been demonstrated. It is possible that psychological factors also influence physiological factors. It has been suggested that chronic musculoskeletal pain should be addressed using a biopsychosocial approach [35]. The experience of pain can be influenced by psychological factors such as stress, depression, and catastrophic thinking [36]. Research suggests that women may be more affected by stress and depression than men [37]. Factors related to physical functioning, such as stress, depression, kinesiophobia, and catastrophization, have also been identified [36]. It is important to note that since the NRS assesses pain and the FAAM assesses function, the psychological factors affecting each score may vary. The longer the disease history, the more chronic and complex the pain [38]. Psychological factors influence chronic pain [39]. The patients who participated in the present study had a long history of the disease; therefore, their pain symptoms probably did not correlate with the blood flow measured in the tissue. PHP is a common overuse syndrome, and the symptoms are often observed in clinical practice. Therefore, developing treatments that combine subjective symptoms and objective data by quantitatively determining lesion repair conditions is desirable. The PSV cutoff value may be clinically useful in understanding tissue repair status.

## 5. Limitations

This study had some limitations. First, using a prospective design intervention to establish how the increase in PSV is altered as pain is relieved is necessary. Second, the association between PSV and imaging findings around the plantar aponeurosis was not evaluated. As mentioned above, imaging findings and pain are associated [25,40]. Therefore, future studies should compare PSV to other imaging findings. Third, the relationship between psychological factors and PSV has not yet been investigated. Psychological factors are associated with chronic pain [39], and investigating how they are related to PSV is necessary. Fourth, the relationship between BMI and blood flow velocity was not assessed because the control and PHP groups were matched for age and BMI. It has been reported that a high BMI is a risk factor for PHP [41]. Understanding the differences in blood flow velocity and pathology in people with a high BMI in PHP may help in understanding the development of PHP in the future. Fifth, the blood flow velocity in the large arteries was not measured. It is possible that this increase in PSV was caused by an increase in the blood flow velocity in the large arteries. However, since there was no correlation between the NRS and PSV in the PHP group (Table 2), we considered the possibility that a redistribution of blood flow due to tissue repair may have occurred.

## 6. Conclusions

In the present study, the PSV of PTA was measured in patients with PHP. The PSV value of the affected side was significantly higher in patients with PHP compared with that of healthy controls. The unilateral cutoff value was 38.2 cm/s. A significant moderate positive correlation was observed between the change in PSV and the change in the NRS score. Future studies are needed to examine changes in PSV over time with long-term interventions.

## Figures and Tables

**Table 1:** Characteristics of the participants in each group.

		PHP Group	Control Group	*p*-Value
Number		23	23	
Age (years)		58.0 ± 16.5	51.3 ± 17.3	0.12
Sex	Male	13 (28.3%)	10 (21.7%)	0.37
	Female	10 (21.7%)	13 (28.3%)	
Height (m)		1.6 ± 0.1	1.6 ± 0.1	0.75
Weight (kg)		59.9 ± 11.5	59.1 ± 11.3	0.84
BMI (kg/m^2^)		22.7 ± 3.3	22.2 ± 3.0	0.87
Blood pressure (mmHg)		128.2 ± 12.4	121.2 ± 24.6	0.23
FAAM ADL score		79.9 ± 16.5	97.5 ± 5.3	<0.001
FAAM Sports score		61.6 ± 27.2	91.8 ± 14.4	<0.001

Data other than Number are presented as mean ± standard deviation. ADL, activities of daily living; BMI, body mass index; FAAM, Foot and Ankle Ability Measure questionnaire; PHP, plantar heel pain.

**Table 2:** Correlation between the peak systolic velocity of the posterior tibial artery and other factors in plantar heel pain.

Factor	R	*p*-Value
NRS score	0.12	0.58
FAAM ADL score	0.21	0.35
FAAM Sports score	0.10	0.67
Blood pressure	0.16	0.43

ADL, activities of daily living; FAAM, Foot and Ankle Ability Measure questionnaire; NRS, Numerical Rating Scale.

**Table 3:** The numerical values and the amount of change in scores before and after the 1-month follow-up in the PHP group (*n* = 18).

Factor	Before Treatment	After Treatment	Amount of Change
PSV (cm/s)	45.0 ± 13.4	39.3 ± 9.1	−5.8 ± 14.5
NRS score	6.5 ± 2.0	3.6 ± 2.1	−2.9 ± 2.8
FAAM ADL score	80.6 ± 17.0	90.0 ± 10.9	9.4 ± 14.1
FAAM Sports score	63.4 ± 25.4	79.7 ± 19.1	16.3 ± 21.9

Data are presented as mean ± standard deviation. ADL, activities of daily living; FAAM, Foot and Ankle Ability Measure questionnaire; NRS, Numerical Rating Scale; PHP, plantar heel pain; PSV, peak systolic velocity.

**Table 4:** The correlation between the change in peak systolic velocity and the change in each factor in the PHP group after 1 month of treatment (*n* = 18).

Factor	R	*p* Value
NRS score	0.47	<0.05
FAAM ADL score	−0.24	0.31
FAAM Sports score	0.02	0.92

ADL, activities of daily living; FAAM, Foot and Ankle Ability Measure questionnaire; NRS, numeric rating scale; PHP, plantar heel pain.

## Data Availability

The datasets relevant to the present study are available on request to the corresponding author.
